# Supplementary material for: Identification of photoperiod-induced specific miRNAs in the adrenal glands of Sunite sheep (Ovis aries)
Source: Front Vet Sci. 2022 Jul 22;9:888207. doi: 10.3389/fvets.2022.888207 (PMC9354845; doi:10.3389/fvets.2022.888207)
Supplement: Supplementary file 1 [file Data_Sheet_1.ZIP › Supplementary file/miRDeep2 file.zip!/miRDeep2 file/Novel_Structure/LP42A2/Novel_21.pdf]

Provisional ID	:	NC_019484.2_37525
Score total	:	1.4
Score for star read(s)	:	-1.3
Score for read counts	:	0
Score for mfe	:	1.1
Score for randfold	:	1.6
Score for cons. seed	:	
Total read count	:	24
Mature read count	:	24
Loop read count	:	0
Star read count	:	0

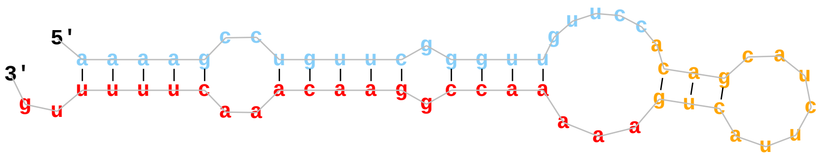

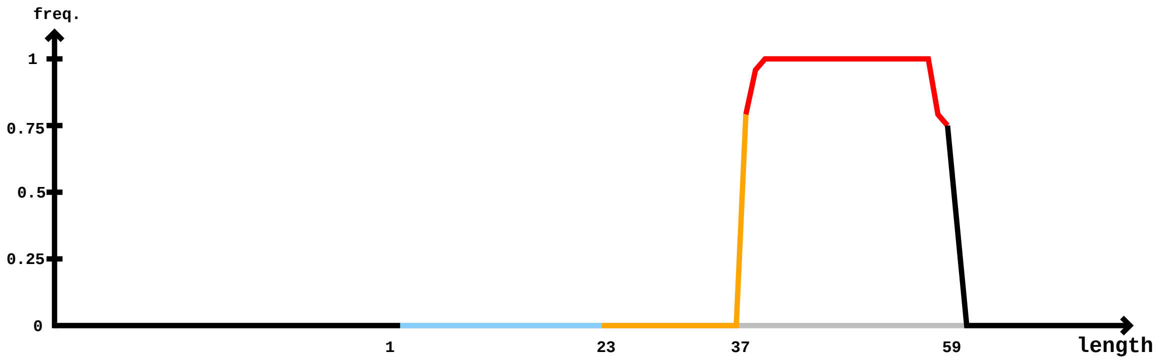

Star Mature

```
5'- cuauuaaaucaaagucauuauauuggguuagccaaaaagccuguucggguuguuccacagcaucuuacugaaaaaccggaacaaacuuuuugguuaaaccaauacaagaa   -3'    exp
    ....................((((((..(((((((((((..(((((.((((......(((.......)))...)))).)))))..)))))))))))..))))))......    reads      mm        sample
    ......................................................................aaaaaccCgaacaaacuuuu....................    3          1         seq
    ......................................................................aaaaaccggaacaaacuuuu....................    1          0         seq
    ......................................................................aaaaaccUgaacaaacuuuuug..................    13         1         seq
    ......................................................................aaaaaccCgaacaaacuuuuug..................    2          1         seq
    .......................................................................aaaaccUgaacaaacuuuu....................    1          1         seq
    .......................................................................aaaaccUgaacaaacuuuuu...................    1          1         seq
    .......................................................................aaaaccUgaacaaacuuuuug..................    2          1         seq
    ........................................................................aaaccUgaacaaacuuuuugg.................    1          1         seq
```
